# The Prevalence of Glaucoma and Its Related Factors in Rural Residents: A Cross-Sectional Study in Jiangxi, China

**DOI:** 10.1155/2021/5551837

**Published:** 2021-05-15

**Authors:** Xiaojun Zhou, Qin Zhu, Jinglin Yi, Jiajunni Li, Qi Li, Jie Kuang, Yuanan Lu, Rui Zhou, Jiayan Chen

**Affiliations:** ^1^School of Public Health & Jiangxi Province Key Laboratory of Preventive Medicine, Nanchang University, Nanchang 330006, China; ^2^Affiliated Eye Hospital, Nanchang University, Nanchang 330006, China; ^3^Department of Public Health Sciences, University of Hawaii at Manoa, Honolulu, HI 96822, USA

## Abstract

**Background:**

This study aims to investigate the prevalence of glaucoma and its related factors among residents aged 40 and over in Jiangxi Province, China, and provide a scientific basis for the prevention and control of glaucoma.

**Methods:**

The cluster sampling method was used to randomly select six townships. Similarly, eight villages were randomly selected from each sample township. A total of 5385 rural residents from 48 villages were collected for a questionnaire survey. A logistic regression model was used to explore the personal behavioral factors related to glaucoma.

**Results:**

Among the 5385 participants, the prevalence rate of glaucoma was 1.4%. The logistic regression model found that alcohol consumption, vegetable consumption, physical exercise, daily reading time, and frequent reading environment were related to glaucoma.

**Conclusion:**

To prevent the occurrence of glaucoma, it is important for rural residents to reduce the frequency of alcohol consumption, increase the frequency of vegetable consumption and physical exercise, control the length of daily reading, and read in a moderately lit environment.

## 1. Introduction

Glaucoma describes a group of eye disorders that cause optic nerve fiber damage and visual field defects. It is one of the leading causes of vision loss and the second blind eye disease in the world after cataract [1, 2]. In addition to increased intraocular pressure (IOP), the causes of glaucoma include abnormal blood flow, oxidative stress, and autoimmune processes. These risks can cause damage to the optic nerve and retinal ganglion cells, resulting in loss of visual field, which is extremely harmful [3]. In 2013, the global prevalence rate of glaucoma in the 40-to-80-year-old population was 3.54%, with a total of 64.3 million patients. The number of global glaucoma patients was estimated to be 111.8 million by 2040 [4, 5]. In 2015, the prevalence rate of glaucoma in China was 2.58% (13.12 million patients), and it is estimated that the number will increase to 25.16 million by 2050 [6]. The growing epidemic situation of glaucoma poses a significant threat to public health.

Recent studies have proved that the prevalence of glaucoma varies significantly depending on the demographics [7]. For example, the Maccabi Glaucoma Study reported that the prevalence of glaucoma was 0.28% in people aged 40–50 and 9.2% in those aged 80 or above, indicating that the prevalence is closely related to age [8]. Another study in China also reported that glaucoma prevalence is higher in the aged, females, and those with a lower level of education [9]. Since the importance of maintaining healthy lifestyle behaviors is vastly mentioned, the relationship between personal behavior and glaucoma has attracted much attention. However, results in different studies have been contradictory. For example, a survey of people in the United States aged 43–84 years showed that alcohol consumption did not affect glaucoma incidence [10]. Inconsistently, the other 12-year follow-up study of African-American women found that alcohol drinking is highly correlated with glaucoma [11]. These contradictory findings should motivate further research in exploring the risk factors of glaucoma.

China has a large population, and the degree of aging is increasing [12], which may increase the risk of developing glaucoma. Therefore, it is necessary to pay attention to the research on the prevalence and related glaucoma factors in China. At present, only few studies in China have reported on the relationship between the prevalence of glaucoma and the demographics. Studies on the prevalence of glaucoma and related personal behaviors are limited with inconsistent results. Since glaucoma risk could be reduced by changing individual behavior, the prevalence of glaucoma and related behavioral factors in China requires further research.

This study aims to investigate the prevalence of glaucoma and related behavioral factors in rural residents over 40 years of age in Jiangxi Province, China. Jiangxi Province (located in central China) is a large agricultural province with a poor economic foundation and is well represented in central China's agricultural inland provinces. This study specifically selected Yifeng County, located in the north-central part of Jiangxi with a middle socioeconomic level. It is representative of rural samples and can represent special population groups living in underdeveloped rural areas. This study explores the influence of individual life behavior factors on the prevalence of glaucoma in rural residents, which is of great significance for further preventing and treating glaucoma.

## 2. Materials and Methods

### 2.1. Study Population

The participants of this study were rural residents aged 40 and above in Yifeng County, Jiangxi Province. The inclusion criteria include (a) residents with permanent residency rights in the area and (b) residents who have reached the age of 40 at the time of the survey, using the date of the survey minus the date of birth as the age accounting standard. The exclusion criteria are (a) unwilling to participate in this study; (b) permanent residency in the local area, but has left the local area for more than 6 months; and (c) suffering from severe mental illness and other cognitive disorders, who were unable to complete this survey.

### 2.2. Sample Size and Sampling Methods

The calculation of the sample size for this study was carried out using the following equation (1) [13]:

(1) $$\begin{array}{c} n=\frac{\left[u\alpha^{2}\times p\times \left(1-P\right)\right]}{\delta^{2}}. \end{array}$$

In this study, the reliability was 95%, the allowable error (*p*) was between 10% and 20%, and *δ* = 15%*P*. Since the global prevalence of glaucoma in the 40-to-80-year-old population is 3.54% [6], the sample size was calculated to be 4652. After grasping the overall framework of the administrative area and population distribution of Yifeng County, the cluster sampling method was used. The sampling process was as follows:

- Six townships were randomly selected from 12 townships under the jurisdiction of Yifeng County.
- Eight villages were randomly selected from each sample township.
- Rural residents over 40 years of age from the 48 villages were collected. Finally, a total of 5385 rural residents were enrolled in this study.

### 2.3. Questionnaire

The questionnaire in this study was adapted from the China Health and Nutrition Survey (2015 edition) [14]. The questionnaire has been validated in Chinese settings. The content of the questionnaire covered three parts: (a) the demographics of the participants, such as gender, age, and household registration. (b) The participants' personal life behavior, such as smoking and drinking. (c) The prevalence of glaucoma. Prevalence means that glaucoma has been diagnosed in medical institutions above the county level. The diagnosis criteria for glaucoma patients followed the guidelines of expert consensus of diagnosis and treatment of primary glaucoma in China (2014) [15]. In this survey, glaucoma was ascertained through self-reported physician diagnosis of glaucoma. For the participants self-reporting glaucoma, the information of medical records was required to be provided.

### 2.4. Data Collection

The investigation team was composed of graduate students, professional ophthalmologists, and professional eye nurses who all were uniformly trained and qualified. Officials from the sample townships, village committees, and village groups participated in the on-site organization and coordination. A temporary fixed survey site was set up in each sampled village, and local residents were organized to conduct the surveys. The survey was completed by face-to-face inquiry. The investigation was conducted from October to December 2018.

### 2.5. Data Analysis

Epidata 3.0 [16] was used for data entry. SPSS 20.0 [17] was used for data analysis. Proportions, means, and standard deviations (SDs) were used for descriptive analysis of data. The *χ*2 test and logistic regression model were used to identify the association between the prevalence of glaucoma and related factors. The significant level was set at *α* = 0.05.

### 2.6. Quality Control

Quality control was performed in this study to ensure proper conduction of the survey: (a) In the research and design stage, a large number of documents were referred to, carefully prepared, and planned. The questionnaire and sampling plan were drawn up by referring to relevant materials. (b) During the on-site investigation stage, the training of investigators was carried out, the purpose and significance of the investigation were clarified, the investigation standards were unified, and the procedures and methods of inquiry were standardized. Strict on-site survey data review standards were established, including the completeness and logical review of questionnaires, and uniform numbering and proper preservation of questionnaires were established. There were detailed instructions for the definition of diet, smoking, physical exercise, etc. (c) A liaison system with the local government was established, administrative power was adopted, and organization and implementation were strengthened. Each sample township and village committee had regular administrative personnel involved in the organization and coordination. (d) There is a strict logical review after data entry, and there were unified regulations for data sorting and variable assignment.

## 3. Results

### 3.1. Characteristics and Current Status of Glaucoma of the Participants

As shown in Table 1, the prevalence of glaucoma among the 5385 participants was 1.4%. There was no statistical difference (*p*=0.332) for the prevalence rates between males (1.3%) and females (1.6%). The prevalence of the four age groups (40–49, 50–59, 60–69, and ≥70 years) was 0.6%, 1.0%, 2.0%, and 1.8%, respectively. The differences in the prevalence among age categories were statistically significant (*p*=0.013). The prevalence rates of glaucoma were 1.7%, 0.8%, 1.5%, and 2.5% for residents with illiteracy, primary school, junior high school, senior high school, and above, respectively. The prevalence of glaucoma was different among residents with different education levels (*p*=0.019). The prevalence of unmarried, married or living together, and divorced or separated or widowed group was 3.3%, 1.3%, and 2.1%, respectively, showing marital status had no statistical impact on the prevalence of glaucoma (*p*=0.131). The prevalence rate of county and town/village residents was 2.7% vs. 1.3%, showing that the prevalence rate of glaucoma in town/village areas was significantly lower than that of county glaucoma (*p*=0.016). The prevalence of national civil servants and public institutions, employees of production enterprises, business service personnel (including transportation), agricultural, forestry and fishery production personnel, unemployed or unemployed, and other workers was 1.0%, 0.4%, 1.2%, 1.8%, and 3.5%, respectively, showing that the prevalence of glaucoma was a statistically significant difference among different occupations (*p*=0.002).

### 3.2. Logistic Regression Analysis of Factors Related to Glaucoma

The results of logistic regression analysis were summarized in Table 2, and the standard for including the independent variable was 0.05 (that is, the significance level *α* < 0.05). Adjusting logistic regression showed that alcohol drinking (”≧1 d/w” vs. “<1 d/w,” aOR = 3.691), eating vegetables (”≧1 d/w” vs. “<1 d/w,” aOR = 0.361), physical exercise (”≧3 d/w” vs. “<3 d/w,” aOR = 0.292), daily reading time (”3-5 h/d” vs. “≤2 h/d,” aOR = 2.094, “≧6 h/d” vs. “≤2 h/d,” aOR = 8.241), and reading environment (“strong light” vs. “moderate light,” aOR = 2.100, “weak light” vs. “moderate light,” aOR = 3.080) were risk factors of glaucoma.

## 4. Discussion

This study shows that the prevalence of glaucoma among rural residents in Jiangxi, China, was 1.4%, which was slightly lower than the prevalence of glaucoma among 40-to-80-year-old in Asian countries in 2013 (3.54%) [18]. It seems that the problem of glaucoma in rural areas in China is not that serious compared to other Asian countries. However, it is noteworthy that limited attention has been paid to glaucoma in rural areas by governments and researchers. Further research is needed to solve this health issue better, thus ensuring the fairness of universal health.

The present study shows that alcohol drinking was one of the risks related to the prevalence of glaucoma. The higher the frequency of alcohol consumption, the higher the risk of glaucoma. In some part, this result supports the conclusion of Wise et al.'s study [11] that excessive drinking may cause glaucoma. It is probably that alcohol drinking was related to the IOP's elevation and contributes to the development of glaucoma [19], suggesting that reducing the frequency of alcohol consumption is needed to prevent glaucoma.

Our study found that adequate amounts of vegetable intake are of benefit to reduce the risk of glaucoma. This result is consistent with the work of Kang et al. [20], indicating that a high intake of green leafy vegetables is related to a low risk of developing glaucoma. Since other studies have shown the positive relationship between low intake of vitamin A and high risk of glaucoma [21], it is necessary to strengthen dietary nutrition education, promote a balanced diet, and develop healthy eating habits in Chinese rural residents.

We found that long daily reading time and a frequent reading under weak or strong light will increase glaucoma risk, indicating that reading habits are significantly related to the occurrence of glaucoma. It may be because the pupils' light reflection is weakened in dim or strong light, which may dilate the pupil, thicken the iris root, and narrow or block the chamber's angle, resulting in increased IOP. Therefore, it is necessary to read under moderate light conditions and reasonably control reading time length. It is also essential to strengthen the health education of eye care in rural residents in China.

This study indicates that the risk of glaucoma in residents who took regular physical exercise (≥3 d/w) is significantly lower. Previous studies have shown that physical exercise may decrease IOP and ocular perfusion (OP), resulting in a low risk of glaucoma. For example, Chen et al. demonstrated that aerobic exercise (jogging) might significantly reduce the choroidal thickness and IOP of glaucoma patients [22]. Yang et al. tested glaucoma patients for low-intensity exercise for 10 minutes and high-intensity exercise for 5 minutes. They found that the patients' IOP decreased significantly after exercise, with an average decrease of 5.72 mmHg [23]. Yokota et al. reported that the average IOP of patients with glaucoma who exercise regularly for more than 30 minutes per week (with variable exercise types and intensities) was 1.5 mmHg lower than that of patients who exercise irregularly [24]. It is well known that lack of physical exercise can increase IOP and/or insufficient OP, leading to optic ganglion cell apoptosis and glaucoma [25]. Therefore, for preventing glaucoma in rural residents, physical exercise should be recommended, and measures should be taken to increase adherence to physical exercise.

There were several limitations in this study. First, this survey was a cross-sectional study. Since glaucoma onset is unknown, it may not be possible to determine personal behavior patterns at the onset of glaucoma. Second, this study only selected one county (Yifeng County) from Jiangxi Province. Although Yifeng (a relatively undeveloped area, with income levels comparable to most rural areas) can represent special population groups living in undeveloped rural areas, further studies in a larger sample are needed. Third, since we tried to understand the general connection between glaucoma and personal behavioral factors, this study did not distinguish between primary open-angle glaucoma and primary angle-closure glaucoma. Fourth, since variables such as personal behavior patterns and prevalence of glaucoma were self-reported, there may be a certain amount of information bias. Although we have made efforts to retrieve the medical records for the participants self-reporting glaucoma, there may have missed cases who have never been diagnosed with glaucoma, which raises concern about the false negatives. However, considering that the survey site is one of the visual impairment surveillance areas in Jiangxi Province and the ophthalmic examinations were conducted frequently, the influence of bias might be reduced.

## 5. Conclusions

In sum, this study demonstrated that excessive alcohol drinking, insufficient intake of vegetables, less physical exercise, weak/intense reading light, and extended daily reading time might be related to an increased risk of glaucoma among rural residents in China. These results laid the foundation for the local health departments to prevent glaucoma. Health education could be strengthened among rural areas to develop healthy behaviors and lifestyles, especially good eye-hygiene practices. Aiming at the risks mentioned above related to glaucoma, effective prevention is expected to reduce the prevalence of glaucoma, maintain the rural residents' health, and improve the rural residents' quality of life.

## Figures and Tables

**Table 1:** The relationship between characteristics of participants and glaucoma (*n* = 5385).

Characteristics	Group	Participant (%)	Glaucoma (%)	Prevalence (%)	*χ* ^*2*^	*p*
Gender	Male	2222 (41.3)	28 (35.9)	1.3	0.940	0.332
	Female	3163 (58.7)	50 (64.1)	1.6		

Age	40∼	817 (15.2)	5 (6.4)	0.6	10.799	0.013
	50∼	1725 (32.0)	18 (23.1)	1.0		
	60∼	1705 (31.7)	34 (43.6)	2.0		
	70∼	1138 (21.1)	21 (26.9)	1.8		

Education	Illiteracy	1909 (35.5)	33 (42.3)	1.7	9.917	0.019
	Primary school	1598 (29.7)	12 (15.4)	0.8		
	Junior high school	1436 (26.7)	22 (28.2)	1.5		
	Senior high school and above	442 (8.1)	11 (14.1)	2.5		

Marital status	Unmarried	61 (1.1)	2 (2.6)	3.3	4.067	0.131
	Married or living together	4610 (85.6)	61 (78.2)	1.3		
	Divorce/Widowed	714 (13.23)	15 (19.2)	2.1		

Residential location	County	481 (8.9)	13 (16.7)	2.7	5.821	0.016
	Town/village	4904 (91.1)	65 (83.3)	1.3		

Career	Civil servant	412 (7.7)	4 (5.1)	1.0	17.042	0.002
	Business	242 (4.5)	1 (1.3)	0.4		
	Production	3042 (56.5)	36 (46.1)	1.2		
	Unemployed	1293 (24.0)	23 (29.5)	1.8		
	Other	396 (7.3)	14 (18.0)	3.5		

Total		5385 (100.0)	78 (100.0)	1.4		

**Table 2:** Logistic regression analysis of factors related to the prevalence of glaucoma (*n* = 5385)^1^.

Variables		Total number (%)	With glaucoma (%)	Unadjusted		Adjusted^1^	
				*p*	OR (95% CI)	*p*	OR (95% CI)
Sleep length	<6 h/d	1647 (30.6)	32 (1.9)	0.091	1.499 (0.938～2.396)	0.261	1.316 (0.816～2.123)
	≥6 h/d	3738 (69.4)	46 (1.2)				
Water consumption	≤1000 ml/d	3354 (62.3)	49 (1.5)	0.719	0.914 (0.561～1.490)	0.860	0.956 (0.584～1.567)
	＞1000 ml/d	2031 (37.7)	29 (1.4)				
Smoking	No	3950 (73.7)	17 (1.2)	0.024	0.511 (0.286～0.914)	0.212	0.631 (0.306～1.302)
	Yes	1435 (26.3)	61 (1.5)				
Alcohol drinking	<1 d/w	3919 (72.8)	38 (1.0)	<0.001	3.341 (2.063～5.411)	<0.001	3.691 (2.250～6.055)
	≧1 d/w	1466 (27.2)	40 (2.7)				
Tea drinking	<1 d/w	4077 (75.7)	60 (1.5)	0.829	0.940 (0.535～1.652)	0.574	0.849 (0.479～1.504)
	≧1 d/w	1308 (24.3)	18 (1.1)				
Regular of meals	<1 d/w	199 (6.4)	3 (1.5)	0.869	1.105 (0.338～3.614)	0.936	0.952. (0.288～3.152)
	≧1 d/w	5186 (93.6)	75 (1.4)				
Taste preference	Moderate	2192 (40.7)	23 (1.0)				
	Salty taste	1747 (32.4)	28 (1.6)	0.247	1.398 (0.793～2.464)	0.188	1.468 (0.829～2.600)
	Light taste	1446 (26.9)	27 (1.9)	0.051	1.770 (0.997～3.145)	0.056	1.763 (0.985～3.154)
Sweet	<1 d/w	3082 (57.2)	49 (1.6)	0.560	0.865 (0.529～1.416)	0.546	0.858 (0.521～1.412)
	≧1 d/w	2303 (42.8)	29 (1.3)				
Greasy food	<1 d/w	3207 (59.6)	48 (1.5)	0.394	0.809 (0.496～1.318)	0.502	0.843 (0.512～1.388)
	≧1 d/w	2178 (40.4)	30 (1.4)				
Vegetables	<1 d/w	193 (3.6)	6 (3.1)	0.033	0.381 (0.157～0.926)	0.027	0.361 (0.146～0.891)
	≧1 d/w	5192 (96.4)	72 (1.4)				
Fruit	<1 d/w	3313 (61.5)	51 (1.5)	0.880	0.962 (0.586～1.582)	0.929	1.023 (0.617～1.697)
	≧1 d/w	2072 (38.5)	27 (1.3)				
Dairy food	<1 d/w	4783 (88.8)	71 (1.5)	0.605	0.804 (0.352～1.839)	0.358	0.674 (0.290～1.564)
	≧1 d/w	602 (11.2)	7 (1.2)				
Reading light	Moderate light	2252 (41.8)	18 (0.8)				
	Strong light	2397 (44.5)	37 (1.5)	0.011	2.109 (1.186～3.752)	0.012	2.100 (1.176～3.748)
	Weak light	736 (13.7)	23 (3.1)	0.001	3.023 (1.587～5.758)	0.001	3.080 (1.607～5.902)
Daily reading time	≤2 h/d	3052 (56.7)	26 (0.9)	0.011	1.984 (1.171～3.360)	0.007	2.094 (1.228～3.570)
	3–5 h/d	2029 (37.7)	33 (1.6)				
	≧6 h/d	304 (5.6)	19 (6.3)	<0.001	7.172 (3.787～13.583)	<0.001	8.241 (4.279～15.874)
Physical exercise	<3 d/w	4290 (79.7)	70 (1.6)	0.014	0.386 (0.180～0.826)	0.002	0.292 (0.132～0.642)
	≧3 d/w	1095 (20.3)	8 (0.7)				
Labor hours	≤3 h/d	3241 (60.2)	48 (1.5)	0.820	0.927 (0.482～1.782)	0.805	1.088 (0.557～2.124)
	3–6 h/d	869 (16.1)	12 (1.4)				
	≧6 h/d	1275 (23.7)	18 (1.4)	0.638	0.872 (0.492～1.544)	0.294	1.388 (0.752～2.561)
Total		5385 (100.0)	78 (1.4)				

^1^Adjust for gender, age, education, marital status, residential location, and career.

## Data Availability

Data can be obtained by contacting the corresponding authors.
